# Suppression of Cancer Cell Stemness and Drug Resistance via MYC Destabilization by Deubiquitinase USP45 Inhibition with a Natural Small Molecule

**DOI:** 10.3390/cancers15030930

**Published:** 2023-02-01

**Authors:** Xiao Tu, Chuncheng Li, Wen Sun, Xi Tian, Qiufu Li, Shaoxin Wang, Xiaoling Ding, Zhen Huang

**Affiliations:** 1 Key Laboratory of Bio-Resource and Eco-Environment of Ministry of Education, College of Life Science, Sichuan University, Chengdu 610000, China; 2 State Key Laboratory of Southwestern Chinese Medicine Resources, Chengdu University of Traditional Chinese Medicine, Chengdu 610000, China; 3 SeNA Research Institute and Szostak-CDHT Large Nucleic Acids Institute, Chengdu 610000, China

**Keywords:** deubiquitinase, cancer stemness, drug resistance, natural product

## Abstract

**Simple Summary:**

Cancer stem cells (CSCs) play significant roles in cancer development, drug resistance and cancer recurrence. Thus, it is of great importance to study and target the mechanism by which CSCs are regulated. On the basis of our investigations, we have discovered that USP45 as a new deubiquitinase of MYC significantly promoted cervical cancer development, stemness and drug resistance. Our findings have established the close connection among USP45, MYC and CSCs. Moreover, we have identified that USP45 can be specifically bound and inhibited by a natural small molecule (α-mangostin), in turn significantly suppressing the USP45-induced stemness and drug resistance of CSCs. On the basis of our USP45 discoveries, a new window has opened for suppressing CSCs development, stemness and drug resistance. Our exciting discovery will attract a broad audience in clinical CSCs target therapy, signaling pathways, natural products, drug discovery and drug development.

**Abstract:**

Cancer stem cells (CSCs) play significant roles in cancer development, drug resistance and cancer recurrence. In cancer treatments based on the CSC characteristics and inducing factors, MYC is a promising target for therapeutic molecules. Although it has been regarded as an undrugable target, its stability tightly regulated by the ubiquitin–proteasome system offers a new direction for molecule targeting and cancer treatment. Herein we report our discoveries in this research area, and we have found that deubiquitinase USP45 can directly bind with MYC, resulting in its deubiquitination and stabilization. Further, USP45 overexpressing can upregulate MYC, and this overexpressing can significantly enhance cancer development, cancer cell stemness and drug resistance. Interestingly, without enhancing cancer development, MYC silencing with shRNA can only suppress USP45-induced stemness and drug resistance. Moreover, we have identified that USP45 can be specifically bound and inhibited by a natural small molecule (α-mangostin), in turn significantly suppressing USP45-induced stemness and drug resistance. Since USP45 is significantly expressed in cervical tumors, we have discovered that the combination of α-mangostin and doxorubicin can significantly inhibit USP45-induced cervical tumorigenesis in an animal model. In general, on the basis of our USP45 discoveries on its MYC deubiquitination and α-mangostin inhibition, suppressing USP45 has opened a new window for suppressing cancer development, stemness and drug resistance.

## 1. Introduction

Although great progress has been made in understanding the mechanisms of cancer development, the disease’s treatments are still unsatisfactory due to many scientific and medical challenges. It has been well demonstrated that cancer stem cells (CSCs) play critical roles in cancer development, tumorigenesis, drug resistance, metastasis and recurrence. CSCs are a small sub-population of tumor cells with the capacity to strongly resist chemotherapy and radiotherapy [1,2,3], and unremoved CSCs can cause residual disease and cancer relapse. It has been observed that CSCs can resist chemotherapy by highly expressing drug efflux devices (ATP-binding cassette transporters) [4,5,6] that remove a range of cytotoxic agents from cells, which reduces the treatment’s effectiveness and allows CSC survival. Likewise, it has been observed that CSCs can resist radiotherapy, causing the enrichment of CD133+ cancer cells (a pivotal sub-population), which drives tumor recurrence [7] in patient xenograft models [8]. The survival and enrichment of CD133+ were attributed to DNA damage repairing by the activation of checkpoint kinase1 (CHK1) and CHK2 [8]. CSCs are distinguished from normal cancer cells by activating signaling pathways (including Wnt/beta-catenin [9], Hedgehog [10], Notch [11], TGF-beta [12] and PI3K/AKT/mTOR [13]) and aberrantly expressing transcription factors (including SOX2, Nanog, OCT4 and MYC [14]), which are CSCs’ hallmarks.

The oncogene MYC, a basic helix-loop-helix (bHLH) transcription factor, play a critical role in regulating various cancer cellular functions, including promoting proliferation, metastasis, stemness and drug resistance [15,16]. Elevated expression of MYC frequently occurs in human cancers and is correlated with tumor aggression and poor clinical outcome [17,18]. MYC stimulates cancer development through activating critical positive cell-cycle regulators, such as cyclins (cyclins D, E, A and B1), CDKs (CDK1, 2, 4 and 6) and E2F transcription factors (E2F1, 2 and 3), or by antagonizing cell-cycle repressors, such as p15, p21 and p27 [19]. Further, MYC can directly promote metastasis and drug resistance [15,20,21]. Clearly, MYC is very important for cancer cell survival and can directly enhance cancer stemness [22]. As targeting MYC has been shown to be very difficult with small molecules, promoting MYC degradation can provide a fine strategy for cancer treatments via CSC suppression [23,24]. 

MYC degradation can be achieved through the ubiquitination and proteasome mechanisms. Ubiquitination is the post-translational reversible modification of proteins that either regulates protein degradation or activity. Ubiquitins are covalently attached to the protein substrates by E1, E2 and E3 enzymes, primarily targeting the substrates for proteasome degradation [25], and ubiquitination can be reversed by deubiquitinases (DUBs). MYC stability is tightly regulated by the ubiquitination system, and MYC is targeted for degradation by the ubiquitin–proteasome pathway after the ubiquitination by SCF^FBXW7^ and several other E3 ligases [26]. Furthermore, ubiquitinated MYC can be stabilized by deubiquitination with DUBs. Interestingly, most of the identified MYC DUBs up to date, including USP22 [27], USP28 [28], USP29 [29], USP36 [30], USP37 [31] and OTUD6A [32], are from one family (Ubiquitin-specific proteases [33], USPs). USPs play key roles in determining the stemness and CSCs’ fate: USP4 stabilizes Twisit1 to promote stemness of lung cancer cells [34], USP8 stabilizes Notch1 to enhance tumorigenesis in breast cancer [35], and USP21 promotes stemness of pancreas cancer cells via Wnt pathway activation [36]. These DUBs (USP22, USP28, USP29, USP36, USP37 and OTUD6A) are observed in stabilizing MYC in cancer cells. However, the direct connections among these DUBs, MYC and cancer stemness are unknown [37,38,39,40,41,42]. Therefore, we have hypothesized that via MYC deubiquitination, USP activity can enhance cancer cell stemness and drug resistance. 

To explore our hypothesis, we have performed bioinformatics analysis on the data from the TCGA [43] and GEO database [44]. Interestingly, we first found that USP45 was highly expressed in cervical cancer tissues and associated with MYC signaling pathways. USP45 complexed with Spindly promotes cancer cell migration [45], and as a critical regulator in DNA damage repair via deubiquitinating ERCC1 it, in turn, stabilizes the XPF–ERCC1 complex promoting survival of cancer cells exposed to the DNA damage agents in cancer treatments [46]. Further, we have discovered that USP45 was a novel deubiquitinating enzyme of MYC, and its overexpressing enhanced cervical cancer stemness and drug resistance. Furthermore, we have found that USP45 inhibition with a natural small molecule promoted MYC degradation and, in turn, reduced MYC-mediated stemness and drug resistance in cervical cancer. Our results highlight USP45 as a critical factor in deciding cervical CSC fate. In conclusion, we have demonstrated that USP45 may play a critical role in enhancing cell survival after cancer treatments, and USP45 inhibition causing MYC destabilization may provide a new strategy for clinical CSC treatments. 

## 2. Methods

### 2.1. Cell Line and Cell Culture

HEK293T (GNHu17), SiHa (TCHu113) and HeLa (TCHu187) cells were purchased from the China Cell Bank. Cells were cultured in DMEM medium (C11995500BT, Gbico, Carlsbad, CA, USA) supplemented with 10% FBS (40130ES76, Yeasen, Shanghai, China) and 100 units/mL penicillin (C0222, Beyotime, Shanghai, China), 100 µg/mL streptomycin (C0222, Beyotime, Shanghai, China). Cells were maintained in the 37 °C incubator with 5% CO_2_ atmosphere.

### 2.2. Plasmids, siRNA and shRNA Information

The plvx-puro vector was used for all protein expressing, and purchased from Shanghai Haijihaoge Biotechnology. The pCMV-Ub and pCMV-Ub-K^0^ vector was purchased from Shanghai Haijihaoge Biotechnology. The pLkO.1-MYC-shRNA was from Cheng-hua Li laboratory (Sichuan University, College of Life Science), and pLKO.1-GFP-shRNA were purchased from Biofeng Biotechnology. The mutants (USP45^C199A^, K^48^ and K^63^) were generated using a QuickMutation™ Plus kit (D0208S, Beyotime, Shanghai, China). All plasmids used in this research were confirmed by DNA sequencing.

pCMV-Ub-K^0^: all lysine (K) in ubiquitin is replaced with arginine (R)

pCMV-Ub-K^48^ or pCMV-Ub-K^63^: only this lysine (K) was retained at the 48th or 63rd position, and the other 6 lysine sites were replaced with arginine (R)

GFP siRNA or shRNA: 5′GCAAGCTGACCCTGAAGTTCAT3′

USP45 siRNA#1: 5′CCUCAUGAUGAAGACUCUU3′

USP45 siRNA#2: 5′GCGAAUACAAGCUAGCAUU3′

### 2.3. Bioinformatics Analyses

Most of the bioinformatics analysis in this research could be implemented via these public database platforms, the functions of which are listed below:

The “TIMER2.0” database (http://timer.cistrome.org/, accessed on 9 May 2021) was utilized for USP45 pan-cancer expression analysis. 

The “GTBAdb” database (http://guotosky.vip:13838/GTBA, accessed on 28 November 2021) was used for Hallmark Gene Enrichment analysis.

The “GEPIA2” database (http://gepia2.cancer-pku.cn/#index, accessed on 15 November 2021) was utilized for survival analysis.

The “GSE63514” dataset from GEO database was processed using R studio, and the mRNA levels of USP45 and MYC in each sample were statistically displayed using Graphpad prism 6.0.

### 2.4. Plasmid Transfection, RNA Interference and Lentiviral Infection

Cells at 50% confluence were transfected with plasmids or siRNA using Lipofectamine 2000 transfection reagent (Invitrogen, Carlsbad, CA, USA), cells were collected at 48–60 h after transfection. 

Recombinant lentiviruses were amplified by co-transfecting packaging plasmids (psPAX2 and pMD2G) and lentiviral expressing plasmids using Lipofectamine 2000 in HEK293T; psPAX2: pMD2G: lentiviral plasmids = 1:1:1. Viruses were collected at 60 h after transfection, and cells at 40% confluence were infected with recombinant lentiviruses in the presence of 10 µg/mL polybrene, followed by 24 h incubation. 

### 2.5. Western Blot

Cells were collected, washed with PBS and re-suspended in RIPA lysis buffer. Equal amounts of total proteins were loaded, separated by SDS-PAGE, transferred to PVDF membranes (ISEQ00010, Millipore, Germany), and hybridized to an appropriate primary antibody and HRP-conjugated secondary antibody for subsequent detection by enhanced chemiluminescence. Western Blot data were quantitated and processed via Image Lab 5.0 software (Bio-Rad, www.bio-rad.com, accessed on 18 May 2018). Original blots can be found at Supplementary Figures S5–S8.

### 2.6. Ubiquitination Assays

HEK293T cells at 50% confluence were transfected with MYC-HA, Ub-Flag and USP45-His using Lipofectamine 2000 transfection reagent (Invitrogen, Carlsbad, CA, USA), cells were treated with MG132 (10 µM) at 42 h and collected at 48 h after transfection. Protein was extracted using a Western/IP lysis buffer (P0013, Beyotime, China). HA-beads were washed twice with lysis buffer and incubated with protein for 6–9 h in a 4 °C shaker. HA-beads were collected, washed twice with PBS and then added with protein loading buffer for 10 min at 95 °C. Ubiquitination was detected by Western Blot.

### 2.7. Tumorsphere Formation Assay

A total of 10^4^ cells were cultured on 6-ultral-low-adherent plates (3471, Corning, Corning, NY, USA) with DMEM medium (C11995500BT, Gbico, Rockville, MD, USA) containing 10% FBS (40130ES76, Yeasen, Shanghai, China), 100 units/mL penicillin (C0222, Beyotime, Shanghai, China), 100 µg/mL streptomycin (C0222, Beyotime, Shanghai, China) for 5 days. Tumorspheres were defined to the diameter of spheres that were greater than 20 μm. Only the image of one visual field was provided, the number of all tumorspheres in each well were counted, normalized and presented to reflect tumorsphere formation ability. In this research, USP45 stable cells were still able to form tumorspheres spontaneously in the normal cell culture condition. For other cells, the special medium (DMEM, 10% FBS, 100 units/mL penicillin, 100 µg/mL streptomycin, 5 mg/mL insulin (Sigma, Taufkirchen, Germany), 2% B27 (Invitrogen, Carlsbad, CA, USA), and 20 ng/mL epidermal growth factor (R&D systems, Tustin, CA, USA) are recommended for tumorsphere formation assay.

### 2.8. Flow Cytometry Assay

CD133+ cell analysis: cells were harvested and washed twice with PBS, then were re-suspended with the staining buffer (00440942, Invitrogen, Carlsbad, CA, USA). The cells were incubated with CD133-PE antibody (12-1338-42, Invitrogen, Carlsbad, CA, USA) for 30 min at 4 °C. The cells were then washed twice with staining buffer and re-suspended using staining buffer, and analyzed by FACS machine (BD LSRfortessa, Franklin Lakes, NJ, USA). 

Apoptosis analysis: cells were treated with doxorubicin (HY-15142A, MCE, Shanghai, China), α-mangostin (HY-N0328, MCE, Shanghai, China), or their combination for 48 h. The cells were harvested, washed with PBS, and incubated with Annexin V-FITC and PI (C1062L, Beyotime, Shanghai, China) for 20 min at room temperature. Then, the cells were analyzed by FACS machine, and Fluorescence-Activated Cell Sorting (FACS) data were processed via Flow Jo software (Ashland, OR: Becton, Dickenson and Company; 2019, www.flowjo.com, accessed on 31 December 2019).

### 2.9. Colony Formation Assay

A total of 2 × 10^3^ cells were cultured on a 6-well plate. After 14 days, the colonies were washed with PBS, treated with 4% paraformaldehyde, and visualized using Diff-Quik stain (G1540, Solarbio, Beijing, China). Only those colonies containing more than 20 cells were counted. 

### 2.10. MTT Assay

Cell viability was analyzed using the MTT agent (C0009S, Beyotime, Shanghai, China). A total of 5 × 10^3^ cells were cultured on the 96-well plates. Cells were treated with doxorubicin, α-mangostin, or their combination for 48 h, then 200 μL of MTT (5 mg/mL) was added to each well. The samples were incubated at 37 °C for 4 h, and then sub-cultured in medium with 200 μL of DMSO (ST038, Beyotime, Shanghai, China). The absorbance of each well was determined at 492 nm. Three independent experiments were performed.

### 2.11. Hoechst 33,342 Assay

A total of 5 × 10^3^ cells were cultured on the 96-well plates. After 12 h, the cells were washed with PBS, treated with Hoechst 33,342 (C0030, Solarbio, Beijing, China), photographed with UV light under a light microscope at high magnification, and the absorbance of each well was determined with a microplate reader at the excitation wavelength (350 nm) and the emission wavelength (461 nm).

### 2.12. Virtual Docking

The USP45 protein structure was simulated using the I-TASSER model [47] and converted to PDBQT format. The α-mangostin structure was downloaded from the PubChem database (Compound ID = 5281650). AutoDock Tools software was used for pretreatment and construction of protein-ligand complexes. AutoDock qVina2 was used for virtual docking between USP45 and α-mangostin. The docking region was framed as the whole USP45 protein, with x = 75.677, y = 78.918 and z = 79.734 axes as the center, and size x = 80, size y = 96 and size z = 76 grids. In virtual docking, 20 docking binding modes were generated, the docking affinities of the top 3 were presented and further analyzed. In addition, virtual docking between USP45 and α-mangostin can be finished directly with CB-DOCK2 [48].

### 2.13. USP45 Expression and Purification

BL21 expressing USP45-His was cultured in TB medium and incubated in a 200 rpm shaker (at 37 °C) until OD = 0.6–0.8. IPTG was added to the medium with the final concentration of 1 mM and was placed in a shaker at 200 rpm at 18 °C overnight. E. coli cells were harvested and resuspended with 10 mL (per 1 g bacteria) of the binding buffer (500 mM NaCl, 50 mM Tris-HCl, 5 mM imidazole, 1 mM PMSF, pH 8.0). After adding into the homogenizer and cracking for 20 min, the supernatant was collected by centrifugation at 20,000× *g*. The impurity particles in the supernatant were removed with 0.22 μm filter, and the protein was purified by AKT-Ni column and eluted by the elution buffer (100 mM NaCl, 20 mM Tris-HCl, 200 mM imidazole). The protein samples were detected using SDS-PAGE and Western Blot.

### 2.14. Alpha-Mangostin/USP45 Affinity Assay (BLI-OCTET K2 Assay)

The USP45 protein samples were concentrated via the ultrafiltration tube and centrifugation, followed by the protein sample transfer to the PBS buffers. Then, the USP45 protein solution and activated biotin (1:100) were incubated together at 4 °C for 4 h. The extra biotin was removed via the ultrafiltration tube and centrifugation, followed by the protein sample transfer to the PBST buffer (0.02% Tween20, pH = 7.4). The interaction between USP45 (50 μg/mL) and α-mangostin (20 μM) was analyzed using BLI-OCTET K2 (Sartorius, Gottingen, Germany).

### 2.15. Assessment of Tumorigenesis via Xenograft Model

All animals were cared for in accordance with the Animal Welfare Act guidelines under an animal protocol approved by Animal Care and Use Committee (Sichuan University, College of Life Science, Chengdu, China). Five-week-old female nude mice (DOSSY experiment animals company, Chengdu, China) were maintained with a regular 12 h light/dark cycle and raised on a standard rodent diet. The different number of cells (5 × 10^4^, 1 × 10^5^, 5 × 10^5^ and 1 × 10^6^) were resuspended in 100 μL of sanitary saline. Mice were randomly divided into 8 groups (*n* = 3 for each group), and xenograft-subcutaneously transplanted with cells. Tumor sizes were measured in half-week intervals. Tumor volume was calculated with the formula 0.5 × L × W^2^, where L and W represent the long and short axes of the tumor. The mice were euthanized at the end of the experiments.

### 2.16. The Combination of α-Mangostin and Doxorubicin Inhibits Tumorigenesis

Five-week-old female nude mice were divided into 4 groups (*n* = 3 for each group) and xenograft subcutaneously transplanted with 1.5 × 10^6^ USP45-induced stem-like SiHa cells. Doxorubicin and α-mangostin were dissolved in corn oil (5% DMSO). The mice were fed via oral gavage every 3 days with corn oil, doxorubicin (5 mg/kg), α-mangostin (40 mg/kg) or their combination. Tumor sizes were measured in each half-week interval. At the end of 28 days, the mice were euthanized, tumors were obtained by skin cutting, and the tumor sizes were measured, weighed and photographed.

### 2.17. Statistical Analysis

GraphPad Prism 6.0 was used for data recording, collection, processing and calculation. All cell-based experiments were performed at least two times in duplicates. Data were presented as means ± SD, and quantitative data were analyzed statistically using Student’s *t*-test to assess their significance.

## 3. Results

### 3.1. USP45 Upregulates MYC Protein Level and Stability

The “TIMER2.0” database [49] was utilized for analyzing the USP45 expression level in various cancers, the result showed that the USP45 expression in partial cancer tissues was higher than normal (Figure S1A). To predict the biological function of USP45 in cancer, Hallmark Gene Enrichment analysis analyzed the effects of USP45 associated with cancer signaling pathways. The results from the “GTBAdb” database [50] suggested that high USP45 expressing activated the cancer signaling, including MYC pathways (Figure S1B,C). The survival analysis from the “GEPIA2” database [51] revealed that only cervical cancer patients with high USP45 expression exhibited poor overall survival (OS) and disease-free survival (DFS) (Figure S2 and Figure S3). These bioinformatics analysis data revealed that USP45 might play a critical role in cervical cancer and activate MYC target signaling pathways. Therefore, we explored the regulatory mechanism of USP45-MYC in cervical cancer. The “GSE63514” dataset [52] showed that USP45 expression in the cancer tissues was elevated, but MYC was not significantly changed in cervical cancer tissues, compared with the normal tissues or stage CIN I-III (Figure S4A,B). There was no correlation between the USP45 and MYC mRNA levels (Figure S4C). Herein, we have hypothesized that USP45 may be a new deubiquitinase of MYC.

**Figure 1:**
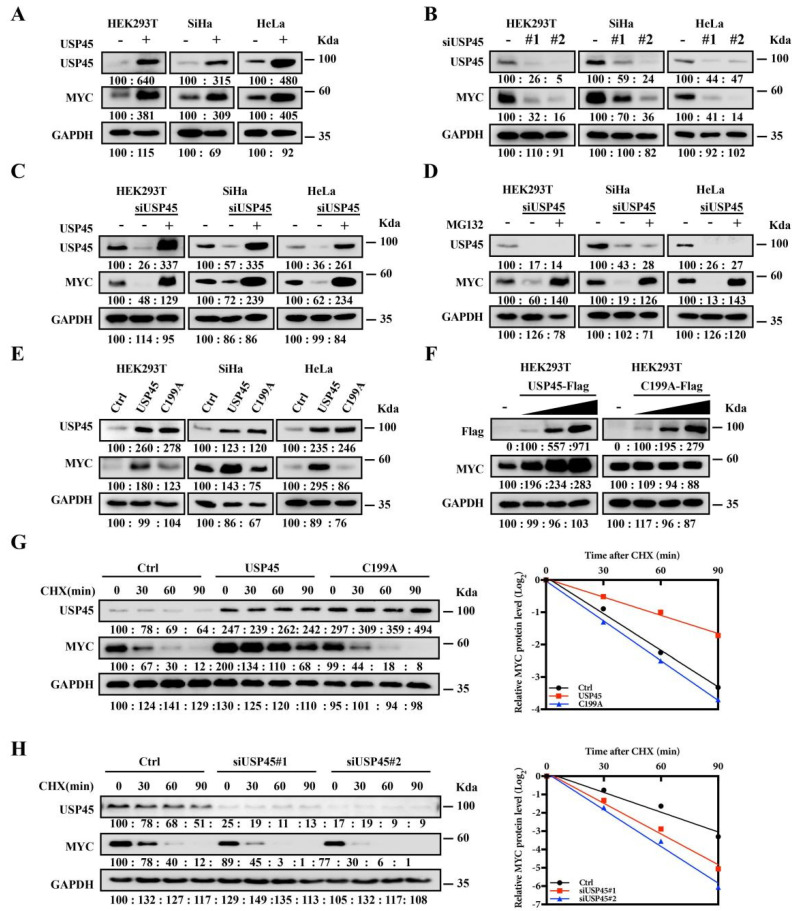
USP45 upregulates MYC protein level and stability. (**A**) Overexpression of USP45 upregulated MYC levels. Cells transfected with USP45 were assayed by Western Blot, plvx-puro vector as the negative control. (**B**) Knocking down USP45 with siRNA reduced the MYC levels. Cells transfected with USP45 siRNA were assayed by Western Blot, GFP siRNA as the negative control. (**C**,**D**) The decreases of MYC with USP45 siRNA were reversed by overexpression of USP45 or addition of a proteasome inhibitor (MG132): (**C**) USP45-expressing plasmid was introduced into cells together with USP45 siRNA, followed by MYC level measurement with Western Blot, plvx-puro and GFP siRNA as the negative control; (**D**) Cells transfected with USP45 siRNA were treated with or without MG132 (20 μM, 12 h), followed by MYC level measurement with Western Blot, GFP siRNA as the negative control. (**E**) USP45 overexpression enhanced MYC level, while its C199A mutant did not. Cells transfected with the control or USP45 (WT or the C199A mutant) were assayed by Western Blot, plvx-puro vector as the negative control. (**F**) USP45 overexpression resulted in MYC elevation in a dose-dependent manner, while its C199A mutant did not. USP45 or C199A with the increased amounts were transfected into cells, and MYC levels were detected, plvx-puro vector as the negative control. (**G**,**H**) USP45 enhanced the stability of MYC: (**G**) SiHa cells transfected with USP45 and C199A were treated with cycloheximide (CHX, 50 μg/mL), and collected at the indicated times for Western Blot, plvx-puro vector as the negative control; (**H**) SiHa cells transfected with USP45 siRNA were treated with cycloheximide (CHX, 50 μg/mL) and collected at the indicated times for Western Blot, GFP siRNA as the negative control. Experiments were conducted at least two times in duplicates.

To investigate the hypothesis, USP45 and its mutant (C199A, the inactive single mutant) were overexpressed in the different cells. We found that USP45 overexpressing significantly upregulated MYC (Figure 1A), and USP45 inhibition with the serval siRNAs significantly decreased MYC (Figure 1B). The MYC decrease by USP45 inhibition with siRNA was reversed by overexpression of USP45 (Figure 1C) or by the addition of the proteasome inhibitor MG132 (Figure 1D). MYC regulation by USP45 was dependent on its DUB activity (Figure 1E), and the USP45 resulted in MYC elevation in a dose-dependent manner (Figure 1F), while its C199A mutant did not have any impact on the MYC level. We treated cells with protein synthesis inhibitor (cycloheximide, CHX) and found that the half-life of MYC was prolonged by USP45 overexpression (Figure 1G), or shortened by USP45 depletion (Figure 1H).

### 3.2. USP45 Interacts with and Deubiquitinates MYC

**Figure 2:**
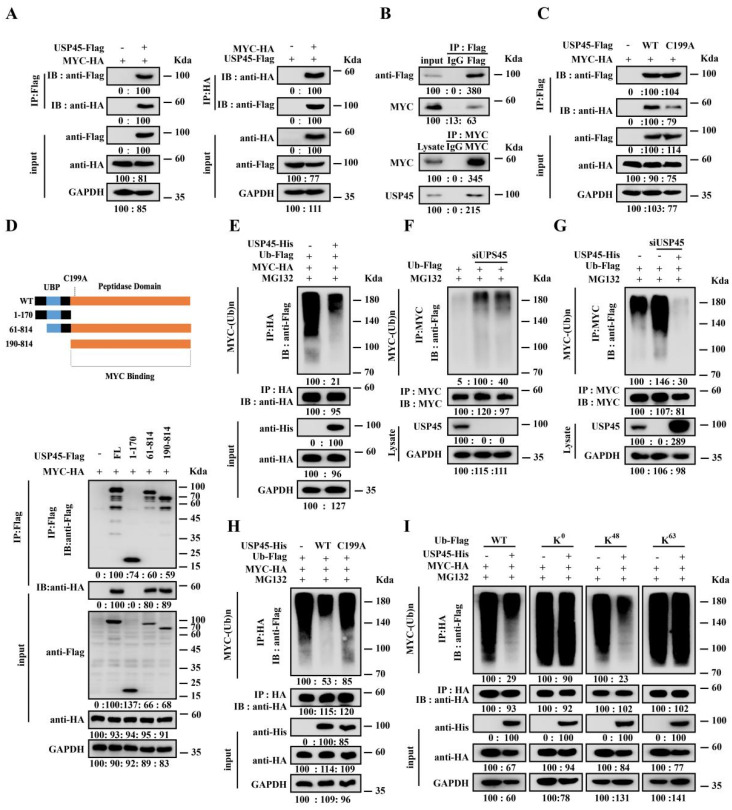
USP45 interacts with and deubiquitinates MYC. (**A**,**B**) USP45 interacts with MYC: (**A**) Co-IP of ectopic USP45 and MYC: HEK293T cells transfected with USP45-Flag and MYC-HA were assayed by co-IP with anti-Flag (left) or anti-HA (right), plvx-puro vector as the negative control; (**B**) Co-IP of ectopic USP45 and endogenous MYC, or endogenous USP45 and endogenous MYC, HEK293T cell lysates were assayed by co-IP with anti-Flag and control IgG (up) or anti-MYC and IgG (down). (**C**) USP45 interaction with MYC was independent from its catalytic activity: HEK293T transfected with USP45-Flag (WT and C199A) were assayed by co-IP with anti-Flag, plvx-puro vector as the negative control. (**D**) The C-terminal domain of USP45 was required for binding to MYC: HEK293T cells transfected by MYC-HA together with control or USP45-Flag (WT or deletion mutants) were assayed by co-IP with anti-Flag, plvx-puro vector as the negative control. (**E**–**H**) USP45 deubiquitinating MYC was dependent on its catalytic activity: HEK293T cells, transfected by the indicated plasmids with USP45 (**E**), USP45 siRNA (**F**), USP45 and USP45 siRNA (**G**), or USP45 and the C199A mutant (**H**), were treated with MG132 for 6 h before harvesting, and the cells were collected for IP experiments, followed by analyzing the ubiquitination of MYC, plvx-puro vector or GFP siRNA as the negative control. (**I**) USP45 deubiquitinated MYC via specifically recognizing the K48 ubiquitin chain: HEK293T cells, transfected with MYC-HA, USP45-His and Ub-Flag (WT, K^0^, K^48^ and K^63^) were collected for the MYC ubiquitination analysis, plvx-puro vector as the negative control. Experiments were conducted at least two times in duplicates.

Co-IP assays demonstrated that USP45 interacted directly with MYC, and the interaction was independent to the USP45 catalytic activity (Figure 2A–C). The deletion analysis demonstrated that the C-terminal domain of USP45 (190–814 domain) mediated its physical interaction with MYC (Figure 2D). Ubiquitination assays demonstrated that USP45 overexpression directly reduced the ubiquitination of MYC (Figure 2E). Conversely, when USP45 was inhibited with siRNA, the ubiquitination of MYC was increased (Figure 2F), and the result was reversed via USP45 overexpression (Figure 2G). Further, the C199A mutant lost the ability to deubiquitinate MYC (Figure 2H). We discovered that the K^48^-link pattern of the ubiquitin chain on MYC was regulated by USP45, while the K^63^-link pattern was not (Figure 2I).

### 3.3. USP45 Promotes Cancer Proliferation, Stemness and Drug Resistance

**Figure 3:**
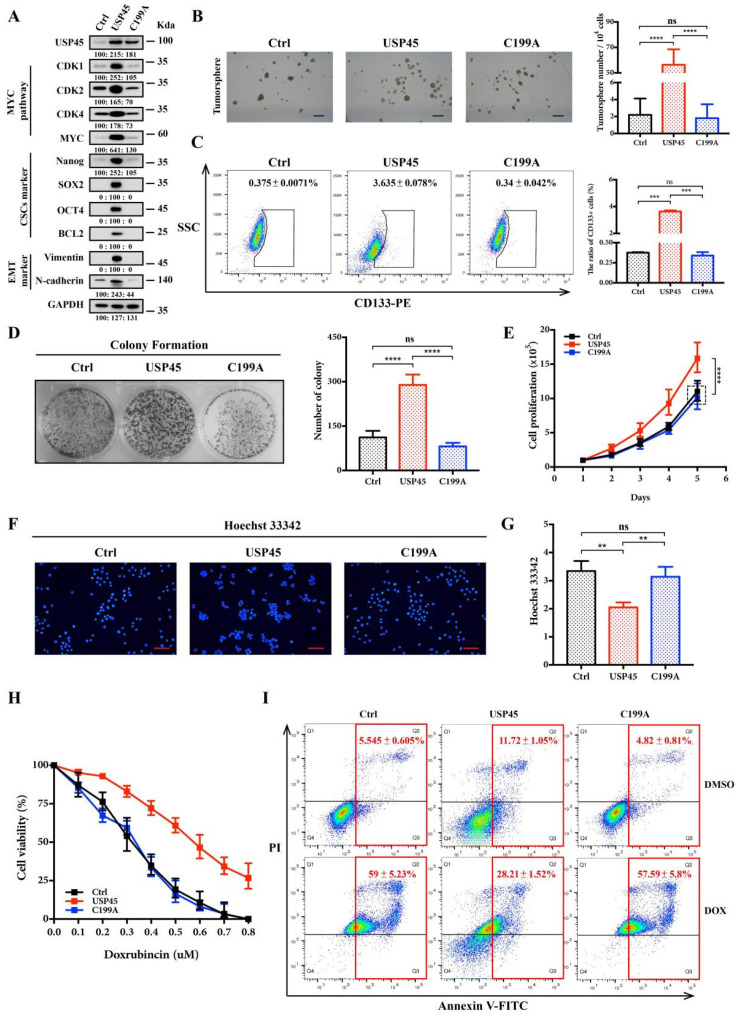
USP45 promotes the proliferation, stemness and drug resistance of cervical cancer cells. (**A**–**E**) SiHa cells stably expressing plvx-puro vector (the negative control), USP45 or C199A were subjected to (**A**) Western Blot analyses, (**B**) tumorsphere formation assay, (**C**) FACS analyses for CD133-stained cells, (**D**) colony formation assay, (**E**) cells proliferation assay and (**F**,**G**) Hoechst 33,342 stain analysis. (**H**) The SiHa cells treated with increasing concentrations of doxorubicin (DOX) were subjected to cell viability assay. (**I**) The SiHa cells treated with 0.5 μM doxorubicin for 48 h were subjected to FACS analyses for Annexin V/PI-stained cells. Respective images and quantitation were shown. Data from independent experiments in duplicates were presented as means ± SD: *p* > 0.05 (no significance/ns), ** *p* < 0.01, *** *p* < 0.001, **** *p* < 0.0001. Scale bar is 100 μm in size. Experiments were conducted at least two times in duplicates.

We established the cervical cancer SiHa cells stably expressing USP45 or C199A. Western Blot assays showed that USP45, but not C199A, upregulated the MYC-targeting proteins (CDK1, CDK2 and CDK4), CSC proteins (Nanog, SOX2, OCT4 and BCL2) and EMT marker proteins (Vimentin and N-cadherin) (Figure 3A). USP45 overexpression enhanced tumorsphere formation (Figure 3B) and increased the population of CD133+ cells (Figure 3C). In addition, the colony formation and proliferation of SiHa cells were significantly increased by USP45 overexpression (Figure 3D,E). Interestingly, we found that USP45 promoted SiHa cells to efflux the Hoechst 33,342 (Figure 3F,G). The cell viability and apoptosis analysis demonstrated that USP45 high expression enhanced drug resistance to doxorubicin (Figure 3H,I). In summary, we demonstrated that USP45 overexpression promoted cancer proliferation, stemness, drug resistance, and transformation of SiHa cells into CSC-like cells.

### 3.4. MYC Is a Downstream Effector in the USP45-Induced Cancer Stemness and Drug Resistance

**Figure 4:**
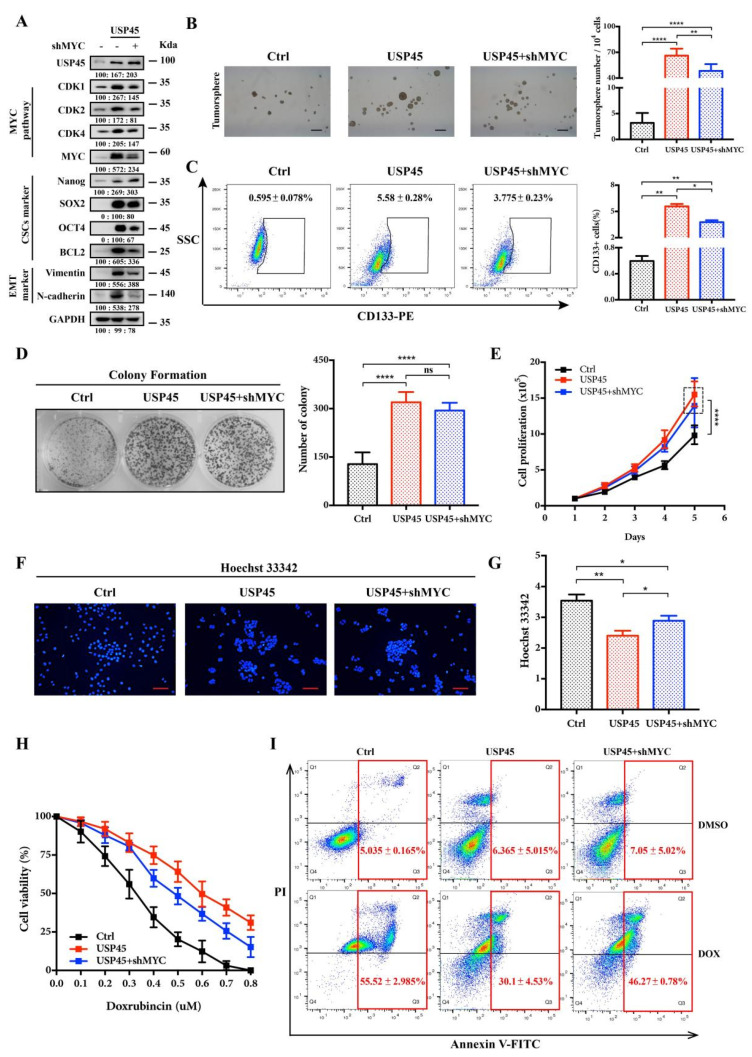
MYC is involved in maintaining the stemness and drug resistance of USP45-mediated cervical CSCs. (**A**–**E**) SiHa cells stably expressing plvx-puro/pLKO.1-GFP-shRNA (the negative control), USP45 and either shMYC were subjected to (**A**) Western Blot analyses, (**B**) tumorsphere formation assay, (**C**) FACS analyses for CD133-stained cells, (**D**) colony formation assay, (**E**) cells proliferation assay and (**F**,**G**) Hoechst 33,342 stain analysis. (**H**) The SiHa cells treated with increasing concentrations of doxorubicin (DOX) were subjected to cell viability assay. (**I**) The SiHa cells treated with 0.5 μM DOX for 48 h were subjected to apoptosis analysis by Annexin V/PI staining. Respective images and quantitation were shown. Data from independent experiments in duplicates were presented as means ± SD: *p* > 0.05 (no significance/ns), * *p* < 0.05, ** *p* < 0.01, **** *p* < 0.0001. Scale bar is 100 μm. Experiments were conducted at least two times in duplicates.

To investigate the causative roles of MYC in the proliferation, stemness and drug resistance of the USP45-induced CSC-like SiHa cells, we performed MYC deletion. In our results, knocking down MYC with shRNA reduced USP45-upregulated proteins (Figure 4A), the tumorsphere number as well as CD133+ cell populations (Figure 4B,C). Interestingly, the colony formation and proliferation of the cells were not changed by knocking down MYC (Figure 4D,E). Further, knocking down MYC inhibited the Hoechst 33,342 efflux capacity of the cells (Figure 4F,G). DOX resistance was significantly reduced by silencing MYC (Figure 4H,I). These results suggested that MYC was a critical downstream effector in USP45-induced cervical cancer stemness and drug resistance, but not proliferation.

### 3.5. Alpha-Mangostin Is an Inhibitor of USP45

**Figure 5:**
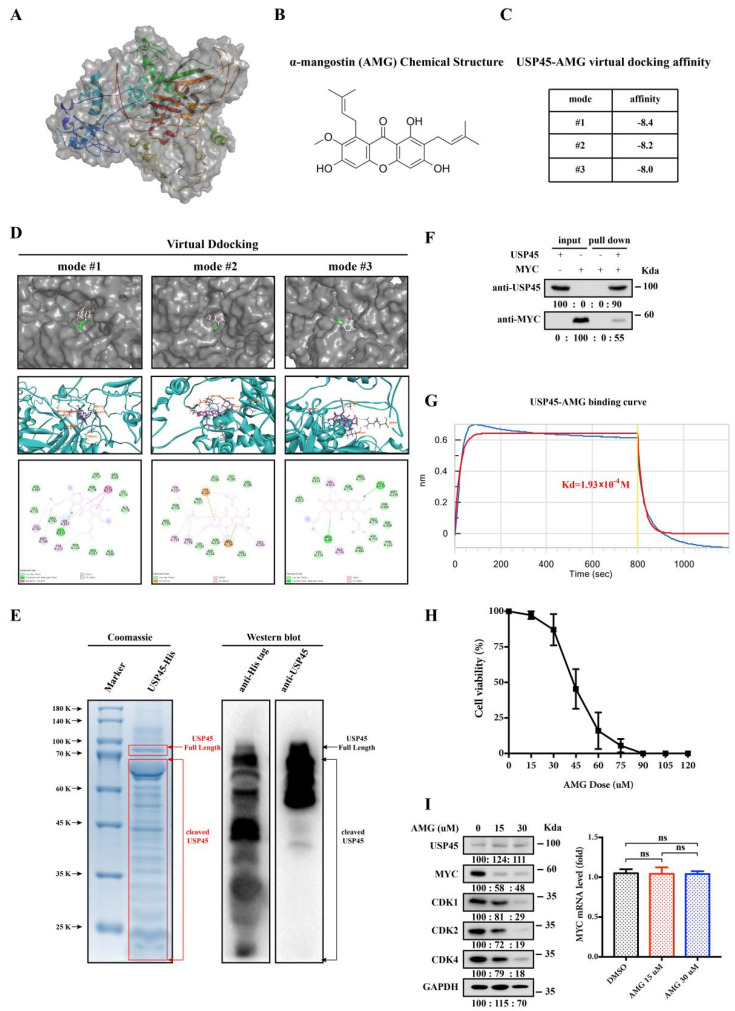
Alpha-mangostin (AMG) may inhibit the deubiquitination-active sites of USP45. (**A**) The structure of USP45 protein was simulated with I-TASSER program. (**B**) AMG chemical structure. (**C**) Top three scores of USP45-AMG virtual docking affinities. (**D**) Interactions between USP45 and AMG: the partial USP45-AMG docking structure (top), 3D diagram (middle) and 2D diagram (bottom) of AMG interaction with the amino acids of USP45. (**E**) USP45 purified from E. coli was analyzed by Coomassie and Western Bolt. (**F**) USP45 purified from E. coli and MYC purified from HEK293T cells were incubated together, followed by pulling down. (**G**) The USP45-AMG affinity was determined using BLI-OCTET K2. (**H**) SiHa cells were treated with different AMG dosages for 48 h, and the cell viability was determined by MTT assay. (**I**) SiHa cells were treated with different dosages of AMG for 48 h and subjected to Western Blot and qPCR analyses, DMSO as the negative control. Experiments were conducted at least two times in duplicates.

We demonstrated that USP45 inhibition via destabilizing MYC suppressed the stemness and drug resistance of cervical cancer cells. As indirect MYC regulation by targeting USP45 can be used as a new strategy for cervical CSC treatments, we screened many USP45 inhibitors. Since the structure of USP45 has not been reported, we firstly used the I-TASSER program to simulate the structure (Figure 5A). Through a series of virtual and experimental screenings, we identified a natural small molecule (α-mangostin, AMG) which interacted with USP45 specifically and tightly bound to the USP45 activity site (Figure 5B–D). To investigate their interaction, USP45 was expressed and purified from E. coli (Figure 5E). The purified USP45 was incubated with purified MYC from HEK293T cells, followed by the pulling down experiments, which demonstrated the direct interaction between USP45 and MYC (Figure 5F). Further, our experimental binding study (BLI-OCTET K2 assay) with USP45 and AMG demonstrated their affinity with Kd of 19.3 μM (Figure 5G). As expected, we observed its strong anticancer effect on cervical cancer cells (Figure 5H) and its downregulation affecting the MYC protein levels without interfering with the mRNA levels (Figure 5I). In summary, we found that the natural small molecule AMG is an USP45 inhibitor.

### 3.6. Alpha-Mangostin Inhibits the USP45-Induced Cancer Stemness, Drug Resistance and Tumorigenesis

**Figure 6:**
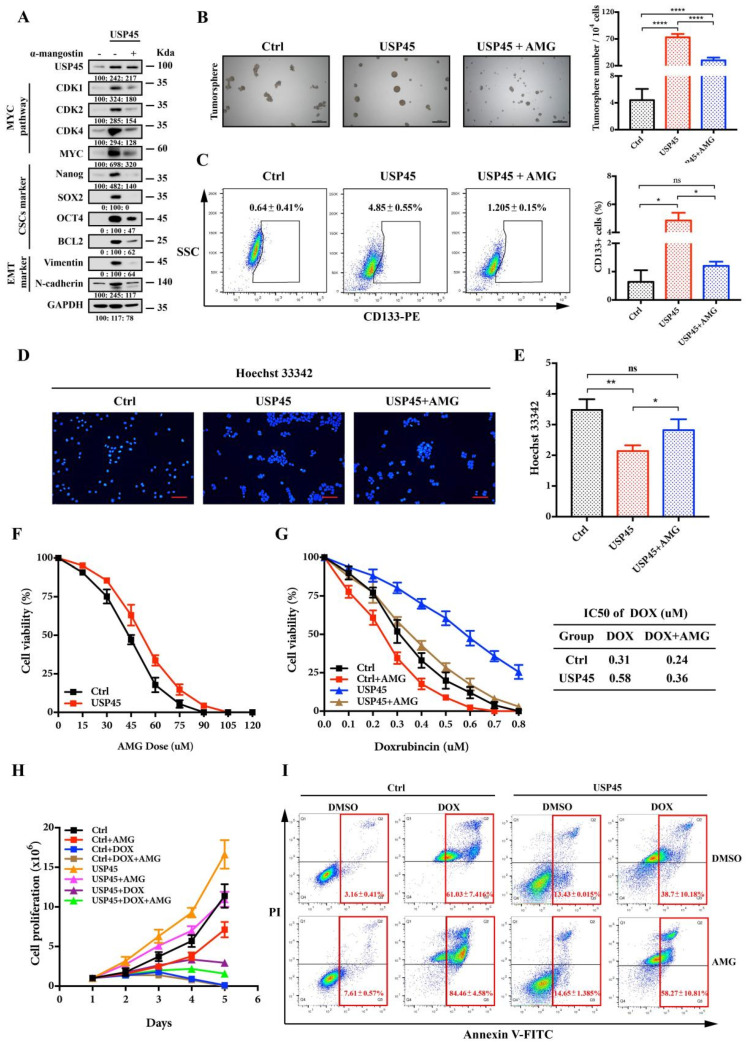
Alpha-mangostin inhibits USP45-induced cervical cancer stemness and drug resistance. (**A**–**D**) SiHa cells stably expressing USP45 were treated with 30 μM α-mangostin (AMG), and cells were collected and subjected to (**A**) Western Blot analysis, (**B**) tumorsphere formation assay, (**C**) FACS analyses for CD133-stained cells and (**D**,**E**) Hoechst 33,342 stain analysis. (**F**) The SiHa cells were treated with increasing concentrations of AMG for 48 h, cells were subjected to MTT cell viability assay. (**G**) The SiHa cells were treated with increasing concentrations of DOX or its combination with 30 μM AMG for 48 h, followed by the MTT cell viability analysis. (**H**,**I**) The SiHa cells were placed on culture plates and treated with 0.5 μM DOX alone, 30 μM AMG alone or their combination, followed by (**H**) the cell proliferation assay and (**I**) the apoptosis assay with Annexin V/PI staining. Respective images and quantitation were shown. Data from independent experiments in duplicates were presented as means ± SD: *p* > 0.05 (no significance/ns), * *p* < 0.05, ** *p* < 0.01, **** *p* < 0.0001. Scale bar is 100 μm. Experiments were conducted at least two times in duplicates.

**Figure 7:**
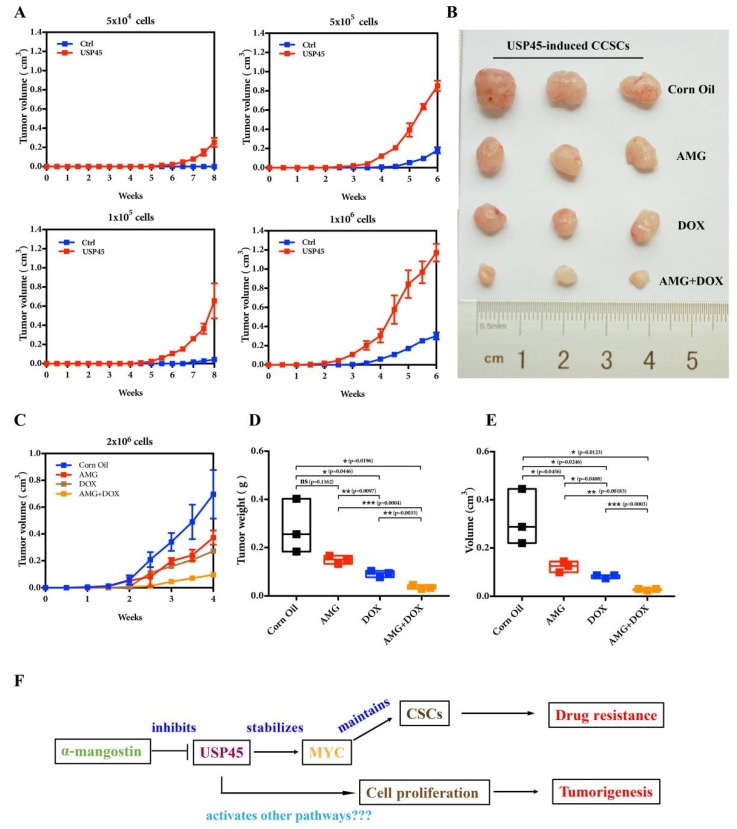
Alpha-Mangostin (AMG) combined with doxorubicin inhibits the tumorigenesis of CSC-like SiHa cells induced by USP45 in the xenograft mouse model. (**A**) The different number of the normal and CSCs-like SiHa cells induced by USP45 were subjected to tumorigenesis assay. (**B**–**E**) The CSCs-like SiHa cells induced by USP45 were subcutaneously implanted into nude mice. Every 3 days for 28 consecutive days, the mice were orally administrated with α-mangostin (40 mg/kg), doxorubicin (5 mg/kg) or their combination. (**B**) The mice were euthanized for tumor analysis. (**C**) Tumor volume analysis during administration. (**D**) Tumor weight. (**E**) Tumor volume. (**F**) The proposed work model of the USP45 system.

To further verify the inhibition of USP45 functions with AMG, we performed the following experiments: Western Blot experiments demonstrated the AMG inhibition on the USP45-induced signaling pathways (MYC targeting, CSC and EMT signaling pathways; Figure 6A). With AMG, the tumorsphere formation, CD133+ cells population and Hoechst 33,342 efflux of USP45-induced CSC-like SiHa cells were significantly inhibited (Figure 6B–E). Interestingly, the cancer cells were not significantly resistant to α-mangostin (Figure 6F). The combination of AMG and DOX exhibited a synergistic effect on inhibiting cancer cell viability and proliferation and promoting apoptosis (Figure 6G–I). Since the USP45-induced CSC-like SiHa cells have strong capacities in tumorigenesis (Figure 7A) and drug resistance, their suppressions were investigated with AMG, DOX and their combination. Our results suggested that their combination had the strongest inhibition on tumorigenesis and drug resistance (Figure 7B–E). In summary, AMG, as the inhibitor of USP45, suppressed cancer development, stemness and drug resistance (Figure 7F).

## 4. Discussion

Our results are consistent with the literature’s studies and demonstrates a new strategy for CSCs treatment. CSCs are intimately involved in cancer recurrence and metastasis, being responsible for the failure of anticancer therapies. It is of great importance to understand the molecular basis of CSC regulation in order to develop new drugs and discover mechanisms of existing drugs for establishing better anticancer strategies.

The discovered direct relationship between USP45 and MYC can be used for suppressing MYC and cancer stemness. Since directly targeting MYC with small molecules and siRNA is difficult, reshaping the regulators of MYC signaling pathways to indirectly control MYC has become a new strategy for cancer treatments. Disrupting MYC-MAX heterodimerization suppresses MYC activity [53]. Further, indirect downregulation of MYC via ubiquitin–proteasome mechanisms is an excellent strategy for targeting CSCs, for instance activating SCF^FBXW7^ [54] and inhibiting deubiquitinase USP28 of MYC suppresses cell proliferation in diverse cancer [55]. The reported deubiquitinases of MYC include USP22 [42], USP28 [37], USP29 [39], USP36 [41] and USP37 [40], and they enhance cancer stemness via BMI1, LSD1 Snail and CEP63 stabilization, respectively. Although these MYC DUBs were reported previously, the direct connection among MYC, DUBs and CSCs was unknown. Clearly, our study established the direct connection between USP45, CSCs and MYC.

Further, we have discovered a USP45 inhibitor (α-mangostin) and a new mechanism for targeting USP45 and CSCs. Alpha-mangostin (AMG), isolated from the mangosteen, is an inhibitor of IDH1^R132H^ (isocitrate dehydrogenase-1 R132H mutant) with a Kd of 2.85 μM [56], and it was reported to interfere with all the major stages of carcinogenesis: initiation, promotion and progression [57]. AMG attenuates stemness and enhances cisplatin-induced stem-like cell death in cervical cancer [58]. However, the mechanisms of its activities remain unknown. Via our structural and functional studies, we have found that AMG can directly inhibit USP45 (with Kd of 19.3 μM), causing MYC destabilization and, in turn, suppressing the stemness and drug resistance of cervical CSCs. This small molecule may be a promising adjuvant drug for clinical CSC treatments.

## 5. Conclusions

On the basis of our investigations, we have discovered that USP45 can directly interact with MYC and is a new deubiquitinase of MYC. Further, we have discovered that increased USP45 expression significantly promoted cervical cancer cell proliferation, stemness and drug resistance, while USP45-enhanced MYC stability facilitated only stemness and drug resistance. These findings have established the close connection among USP45, MYC and CSCs. Interestingly, decreased USP45 activity by AMG inhibition significantly suppressed cancer cell proliferation, stemness and drug resistance. In general, on the basis of our USP45 discoveries on its MYC deubiquitination and α-mangostin inhibition, suppressing USP45 has opened a new window for suppressing cancer development, stemness and drug resistance.

## Data Availability

Not applicable.

## Supplementary Materials

The following supporting information can be downloaded at: https://www.mdpi.com/article/10.3390/cancers15030930/s1, Figure S1: USP45 pan-cancer expression and function analysis, Figure S2: Overall survival of cancer patients was stratified by USP45 expression levels, Figure S3: Disease-free survival of cancer patients was stratified by USP45 expression levels, Figure S4: The “GSE63514” dataset was used to analyze clinical correlation between USP45 and MYC expression levels, Figure S5: Western Blot original data for Figure 1, Figure S6: Western Blot original data for Figure 2A–C, Figure S7: Western Blot original data for Figure 2E–I, Figure S8: Western Blot original data for Figure 3A, Figure 4A, Figure 5E–F,I, Figure 6A.
