# Supplementary material for: Suppression of Cancer Cell Stemness and Drug Resistance via MYC Destabilization by Deubiquitinase USP45 Inhibition with a Natural Small Molecule
Source: Cancers (Basel). 2023 Feb 1;15(3):930. doi: 10.3390/cancers15030930 (PMC9913288; doi:10.3390/cancers15030930)
Supplement: Supplementary file 1 [file cancers-15-00930-s001.zip › cancers-2116900-supplementary.pdf]

cancers

MDPI

Article

Supplementary Materials: Suppression of Cancer Cell Stemness and Drug Resistance via MYC Destabilization by Deubiquitinase USP45 Inhibition with a Natural Small Molecule

Xiao Tu [1,†], Chun-Cheng Li [1,†], Xi Tian [1], Shao-Xin Wang [1], Wen Sun [1], Qiu-Fu Li [1], Xiao-Ling Ding [1] and Zhen Huang [1,2,3,*]

[1] Key Laboratory of Bio-Resource and Eco-Environment of Ministry of Education, College of Life Science, Sichuan University, Chengdu 610000, China
[2] State Key Laboratory of Southwestern Chinese Medicine Resources, Chengdu University of Traditional Chinese Medicine, Chengdu 610000, China
[3] SeNA Research Institute and Szostak-CDHT Large Nucleic Acids Institute, Chengdu 610000, China
* Correspondence: huang@senaresearch.org; Fax: +86-028-8550-2629
† These authors contributed equally to this work.

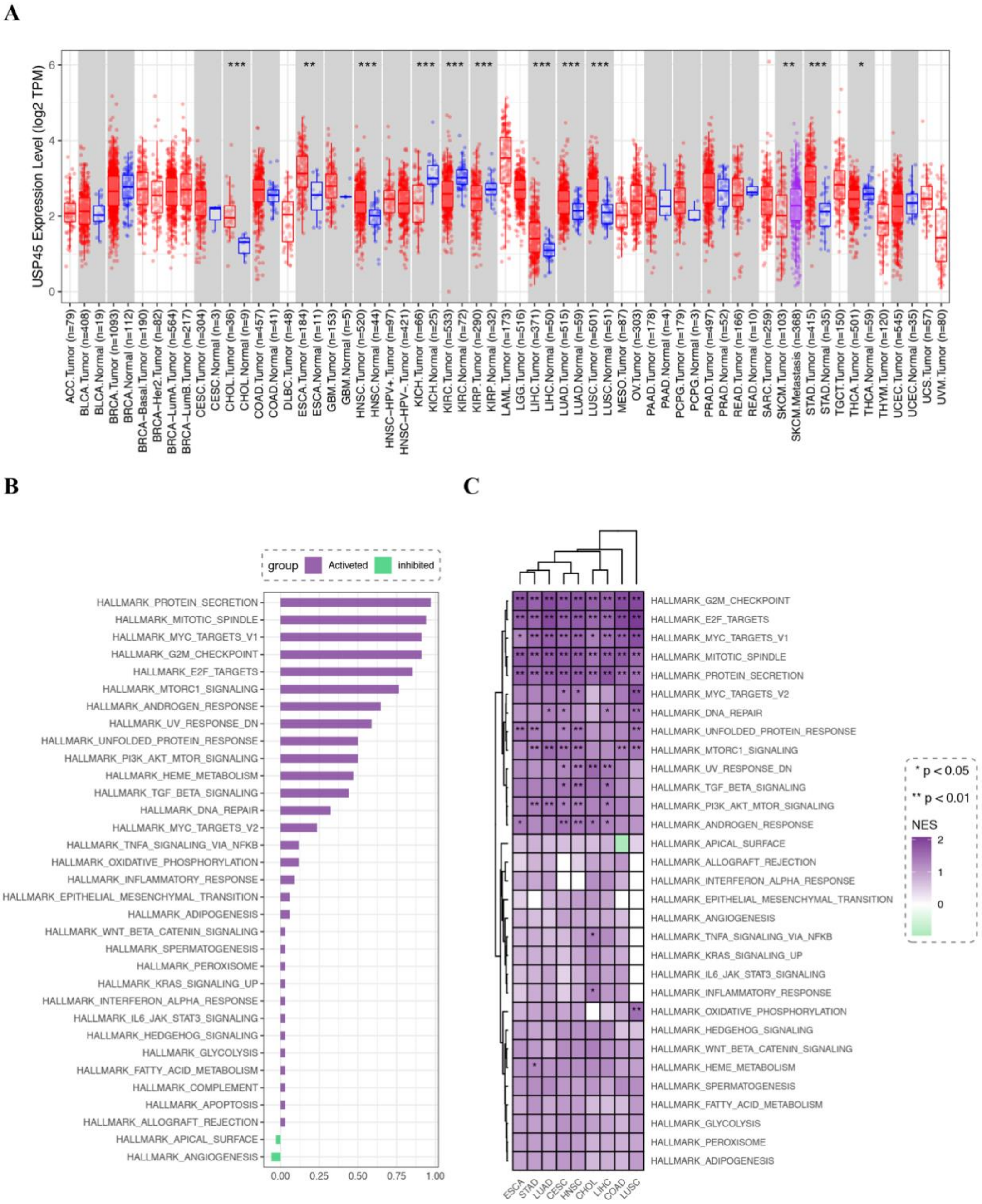

Figure S1. USP45 pan-cancer expression and function analysis. (A) The "TIMER2.0" database was utilized for the USP45 expression level analysis, the USP45 mRNA levels were shown in various cancer. The abbreviation of the types of tumors with high USP45 expression are shown below, and other cancers refer to the TCGA database： CESC (Cervical squamous cell carcinoma and endocervical adenocarcinoma); CHOL (Cholangiocarcinoma); COAD (Colon adenocarcinoma); ESCA (Esophageal carcinoma); HNSC (Head and Neck squamous cell carcinoma); LIHC (Liver hepatocellular carcinoma); LUAD (Lung adenocarcinoma); LUSC; (Lung squamous cell carcinoma); STAD (Stomach adenocarcinoma). (B-C) These cancers with USP45 high expression in Fig. S1A were used to analyze the effects of USP45 associated with cancer signaling pathways: The "GTBAdb" database was used for Hallmark Gene Enrichment Analysis: (B) all cancers, (C) each individual cancer.

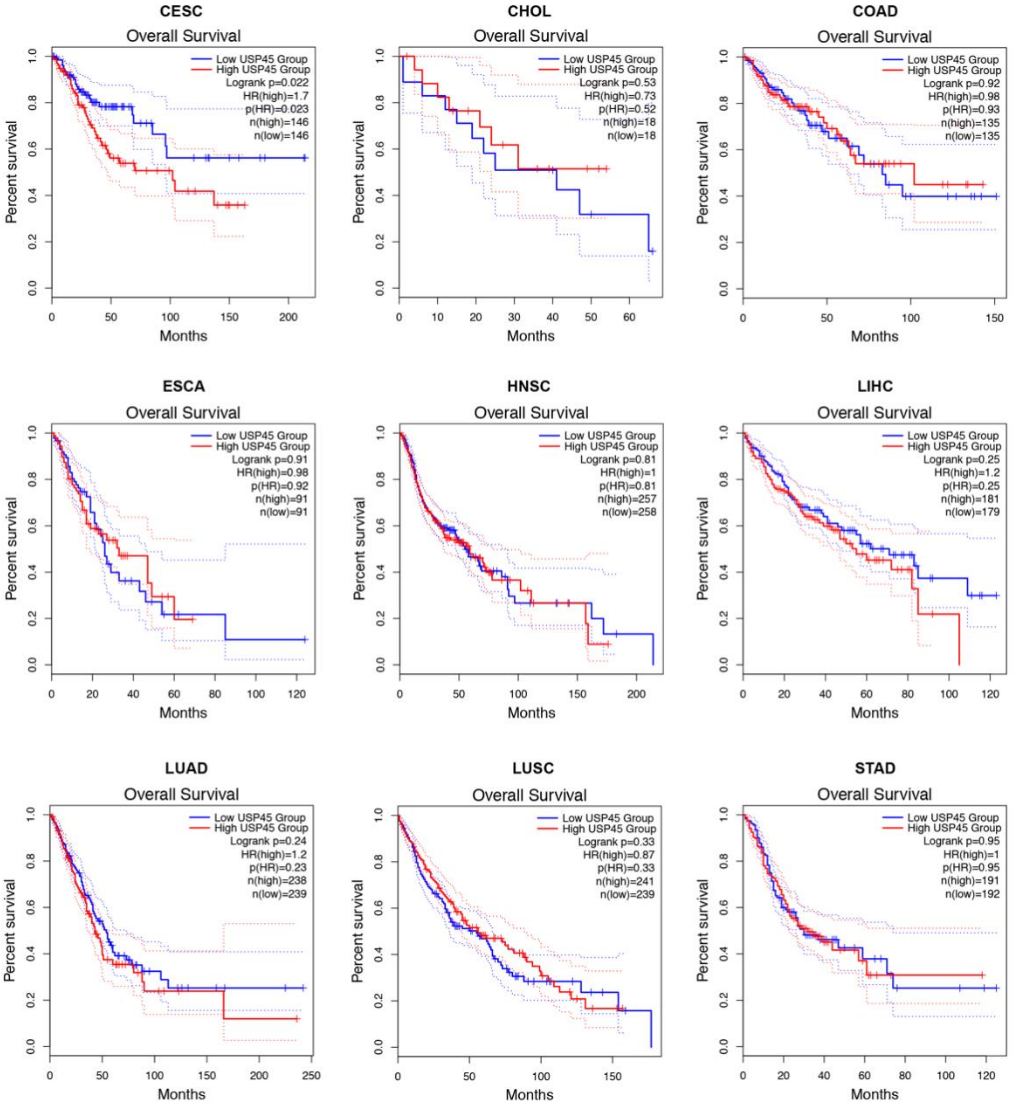

Figure S2. Overall survival of cancer patients was stratified by USP45 expression levels. USP45 expression levels were shown to correlate with the patients' overall survival. The abbreviation of the types of tumors with high USP45 expression are shown below: CESC (Cervical squamous cell carcinoma and endocervical adenocarcinoma); CHOL (Cholangiocarcinoma); COAD (Colon adenocarcinoma); ESCA (Esophageal carcinoma); HNSC (Head and Neck squamous cell carcinoma); LIHC (Liver hepatocellular carcinoma); LUAD (Lung adenocarcinoma); LUSC; (Lung squamous cell carcinoma); STAD (Stomach adenocarcinoma).

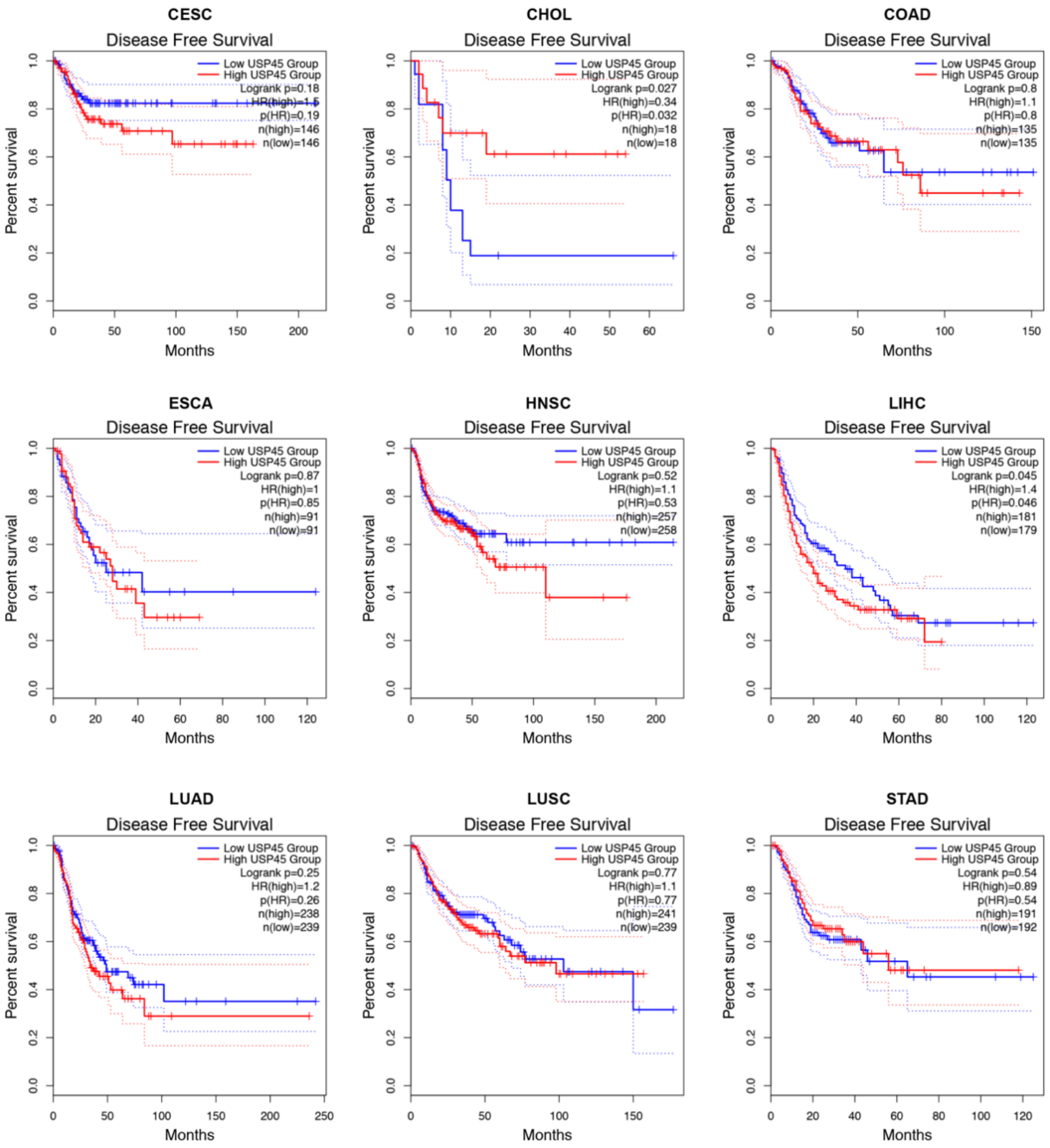

Figure S3. Disease free survival of cancer patients was stratified by USP45 expression levels. USP45 expression levels were shown to correlate with the patients' disease free survival. The abbreviation of the types of tumors with high USP45 expression are shown below: CESC (Cervical squamous cell carcinoma and endocervical adenocarcinoma); CHOL (Cholangiocarcinoma); COAD (Colon adenocarcinoma); ESCA (Esophageal carcinoma); HNSC (Head and Neck squamous cell carcinoma); LIHC (Liver hepatocellular carcinoma); LUAD (Lung adenocarcinoma); LUSC; (Lung squamous cell carcinoma); STAD (Stomach adenocarcinoma).

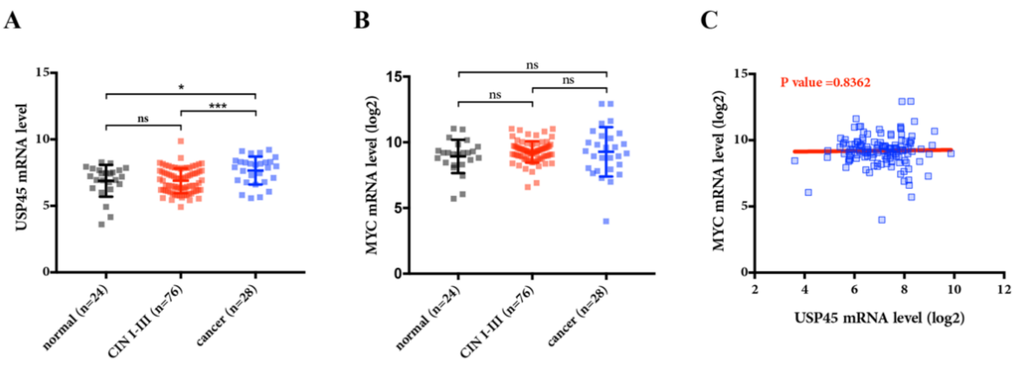

Figure S4. The "GSE63514" dataset was used to analyze clinical correlation between USP45 and MYC expression levels. The "GSE63514" dataset from GEO database was processed by R studio, and the mRNA levels of USP45 and MYC in each sample were statistically displayed by Graphpad prism 6.0. (A) Expression levels of USP45 in different stages of cervical cancer. (B) Expression level of MYC in different stages of cervical cancer. (C) Correlation between USP45 and MYC expression levels.

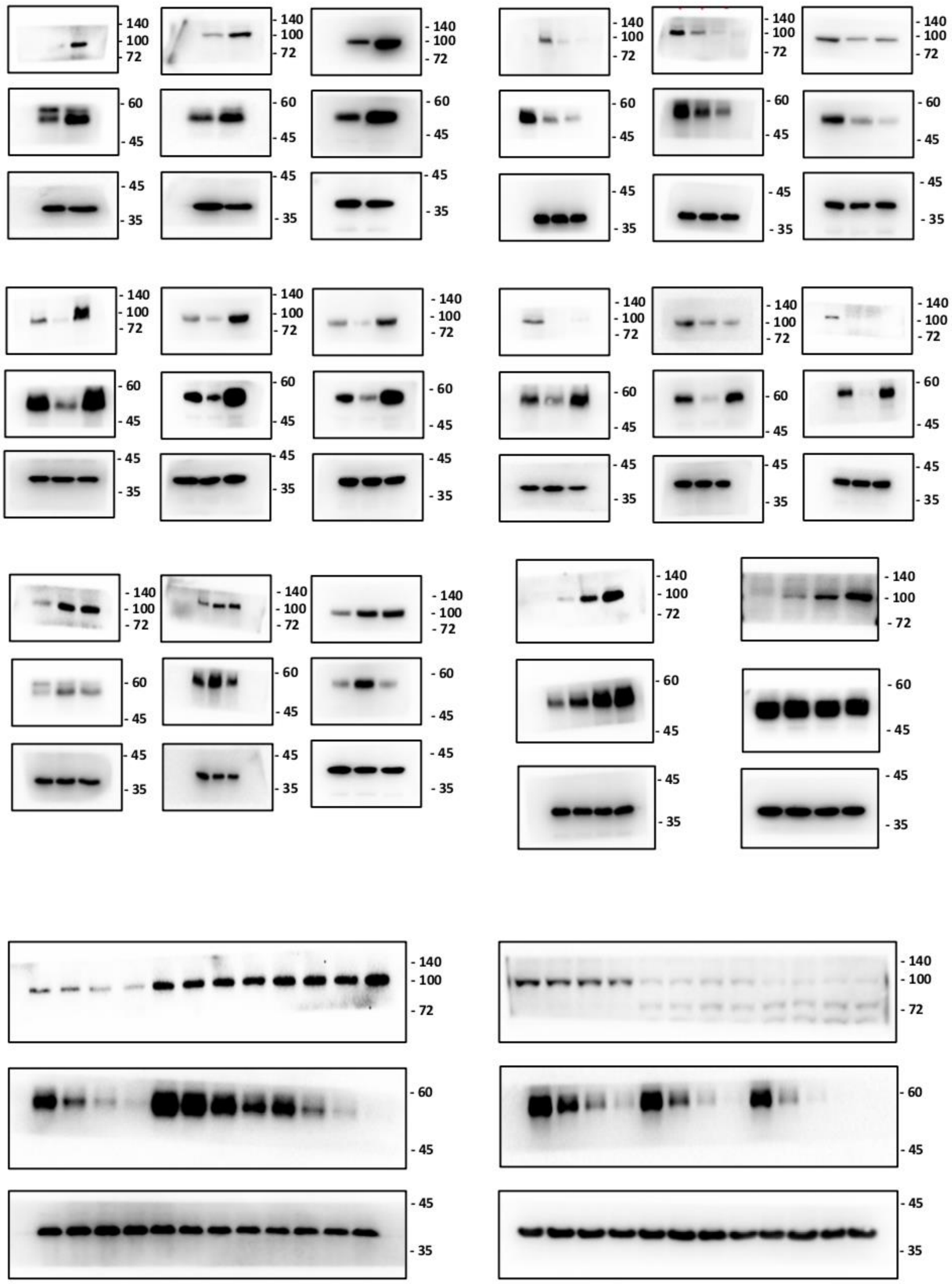

Figure S5. Western Blot original data for Figure 1.

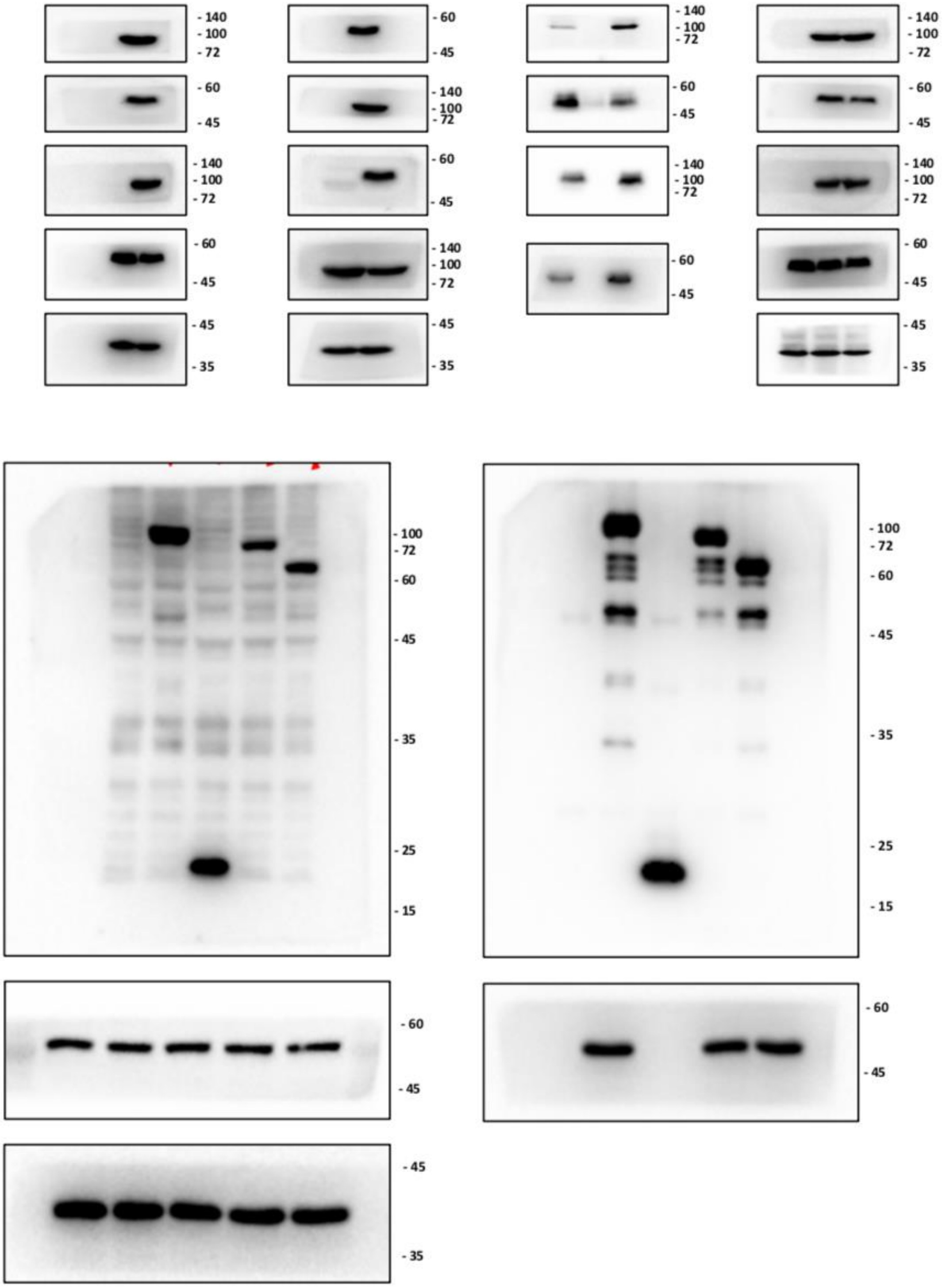

Figure S6. Western Blot original data for Figure 2A–C.

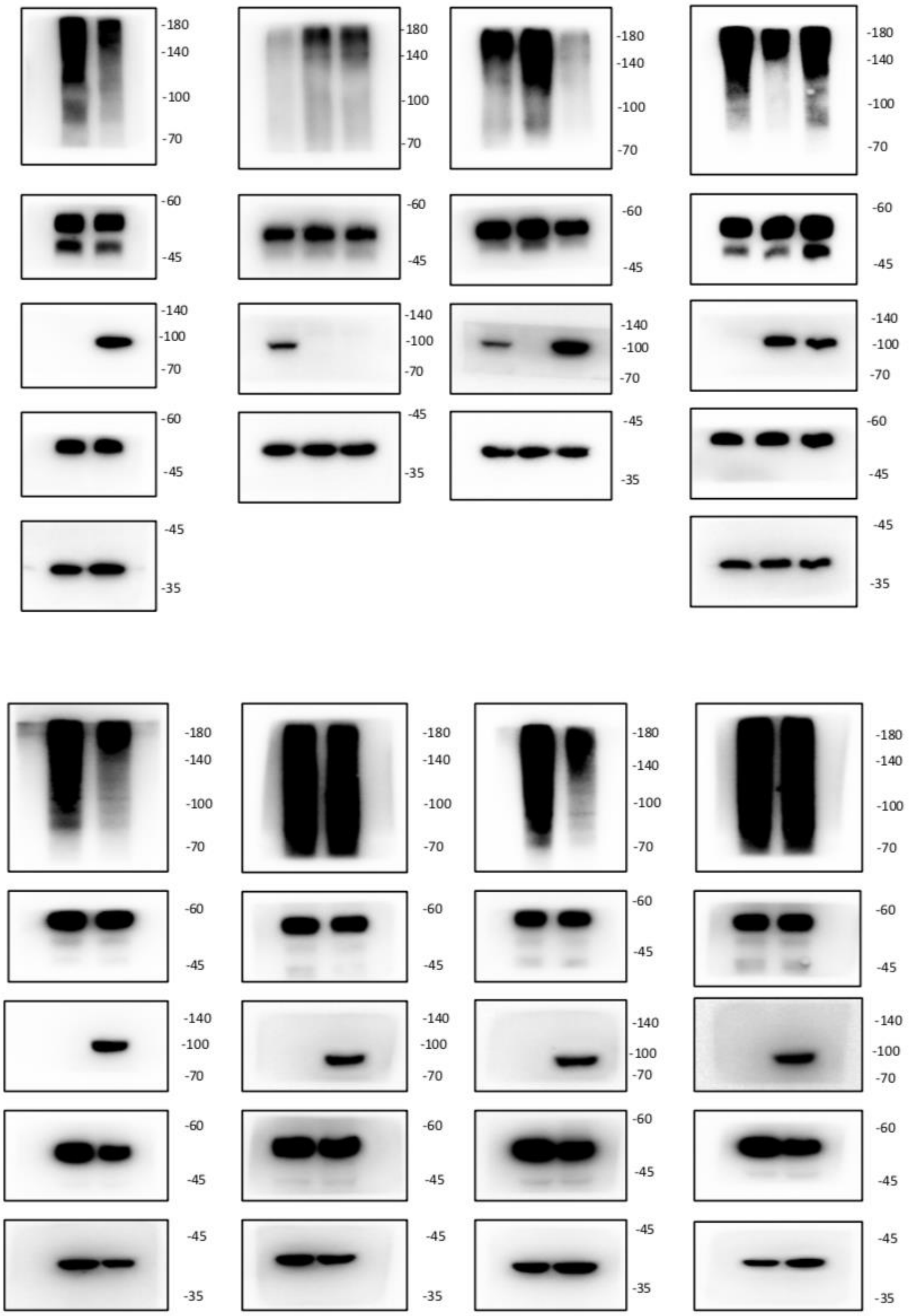

Figure S7. Western Blot original data for Figure 2E–I.

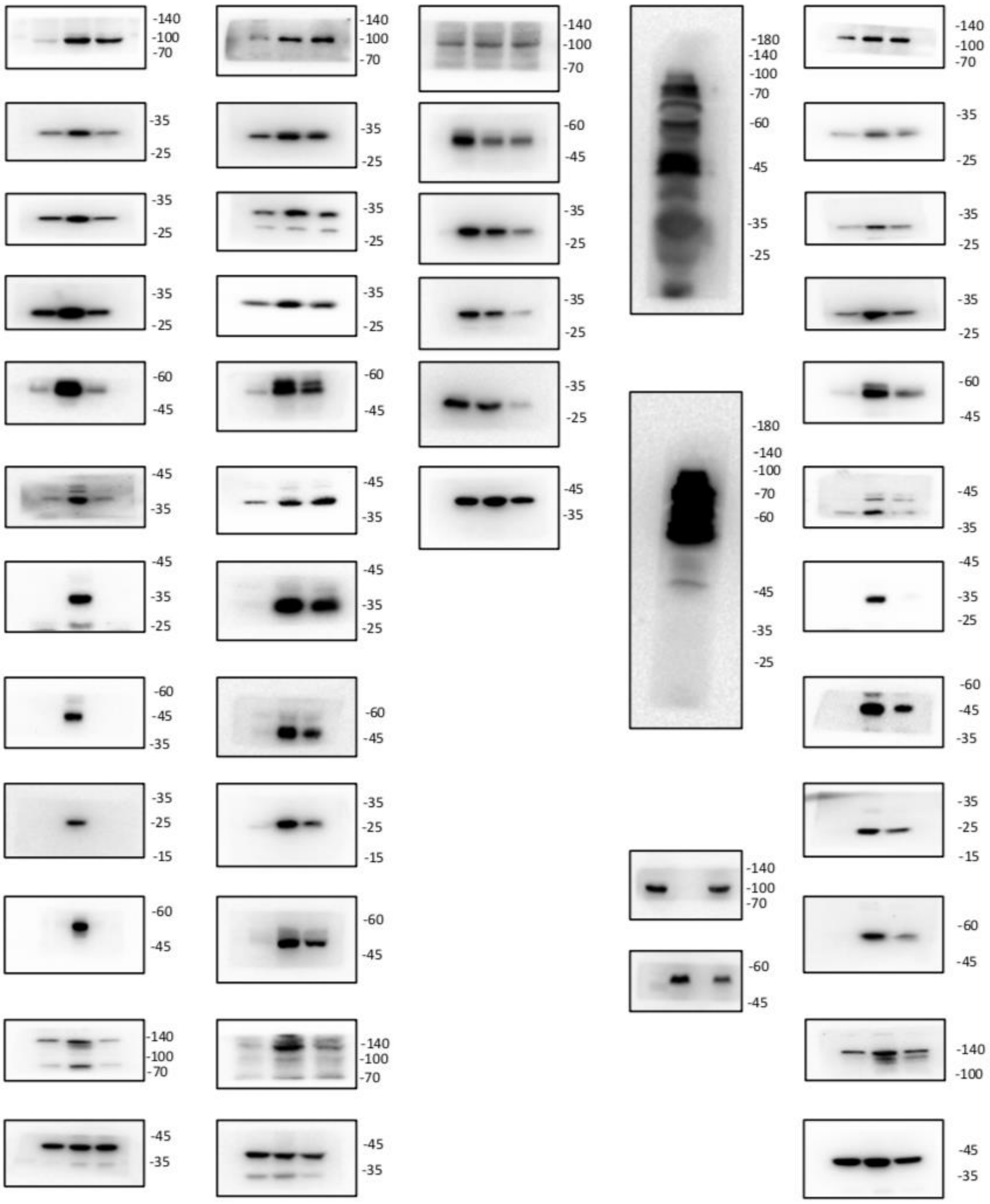

Figure S8. Western Blot original data for Figure 3A, 4A, 5E–F, 5I, 6A.
